# Sentinel lymph node detection in gastric cancer using a dual tracer (Superparamagnetic iron oxide and methylene blue): a prospective study with histological and OSNA validation

**DOI:** 10.1038/s41598-026-43345-7

**Published:** 2026-03-19

**Authors:** Raquel Escalera-Pérez, Carlos Medina-Achirica, Francisco J. García-Molina, Juan J. Del Rio-Ignacio, Carolina Lagares-Franco, Angel Estella, Francisco A. Mateo-Vallejo

**Affiliations:** 1 Surgery Department, University Hospital of Jerez Frontera, Jerez de la Frontera, Spain; 2 Pathology Department, University Hospital of Jerez Frontera, Jerez de la Frontera, Spain; 3 Department of Statistics and Operations Research, University of Cádiz, Cádiz, Spain; 4 Institute for Research and Innovation in Biomedical Sciences (INiBICA), University of Cádiz, Cádiz, Spain; 5 Intensive Care Department, University Hospital of Jerez Frontera, Jerez de la Frontera, Spain; 6 Medicine and Surgery Department, INiBICA University of Cádiz, Cádiz, Spain

**Keywords:** Stomach Neoplasms, Sentinel Lymph Node Biopsy, Methylene Blue, Iron, Nucleic Acid Amplification Techniques, Cancer, Gastroenterology, Medical research, Oncology

## Abstract

Gastric cancer (GC) remains an oncological challenge, with lymph node involvement being a major prognostic factor. Sentinel lymph node (SLN) mapping may improve staging and reduce morbidity. This study evaluated a dual-tracer technique (superparamagnetic iron oxide: SPIO and methylene blue) for ex vivo detection; assessed one-step nucleic acid amplification (OSNA) versus conventional histology; and applied OSNA-pooling analysis in non-SLN. Prospective study of patients undergoing curative gastrectomy with lymphadenectomy (2017–2022). SLNs were identified in fresh specimens by Sentimag^®^ and/or macroscopic staining. Each lymph node was bisected, with one half analyzed by hematoxylin–eosin (H&E) and the other by OSNA. Non-SLN were assessed using pooled OSNA to enhance diagnostic performance. Diagnostic performance metrics and agreement (Cohen’s κ) were calculated. Survival was estimated by Kaplan–Meier. Thirty-eight patients were included (mean age 64.3 years; 58% male); 71.1% received neoadjuvant therapy. SLN detection rate was 84.2% (32/38), with 80 SLNs identified. The dual-tracer approach demonstrated high sensitivity (93.1%) and moderate specificity (44.4%), resulting in an accuracy of 81.6%. Tracer concordance was moderate (κ = 0.42; *p* < 0.007). OSNA showed good agreement with H&E (sensitivity 80%, specificity 87%), and better performance for micrometastases. The pooling analysis evaluated 1155 non-SLN simultaneously; this novel strategy showed good concordance with conventional histology. After a median follow-up of 53 months, OSNA and H&E status were associated with overall survival in exploratory analyses; however, these findings should be interpreted with caution given the limited sample size and ex vivo proof-of-concept design. Combining SPIO and methylene blue is technically feasible for ex vivo SLN mapping. OSNA complements histology, and its pooled use represents an innovative methodological approach. These results are preliminary and hypothesis-generating; larger prospective in vivo studies are required before clinical applicability can be evaluated. Dual-tracer sentinel node mapping in gastric cancer proved feasible and accurate. OSNA complemented histology, pooled analysis improved detection efficiency, and neoadjuvant therapy did not affect diagnostic performance.

## Introduction

Gastric cancer (GC) is among the most prevalent malignancies worldwide and represents a major health problem. In 2022, it ranked fifth among solid tumors for both incidence and mortality. In Europe, 135,610 new cases were reported that year, representing 14% of all cancer diagnoses, while 95,431 deaths were attributed to GC (14.5% of cancer mortality)^1,2^.

The high morbidity and mortality rate represents a major health problem. Lymph node involvement is among the most relevant prognostic factors, and its extent correlates directly with overall survival and recurrence rates^3^. Several studies have demonstrated that D2 lymphadenectomy or more extensive procedures increase in-hospital mortality, overall morbidity, operative time, reoperation rate, and length of hospital stay^4,5^. Therefore, achieving an appropriate balance between oncological radicality and perioperative safety is essential.

In Western series, centralization of GC surgery has been associated with improved lymphadenectomy quality, better overall outcomes, and higher rates of so-called “textbook outcomes”^6^. However, centralization is not always feasible, and outcome heterogeneity persists across lower-volume centers. In this setting, strategies aimed at standardizing nodal staging and lymphadenectomy, such as SLN mapping combined with molecular assessment using OSNA could contribute to improving surgical quality and reducing variability in oncological outcomes.

In this context, current surgical trends are moving toward personalized medicine, with surgical strategies tailored according to an individualized assessment of lymph node involvement. Against this background, sentinel lymph node (SLN) mapping potentially allows more accurate estimation of nodal spread while minimizing morbidity without compromising therapeutic efficacy^7^. In the present study, SLN detection was performed ex vivo after specimen extraction as a proof-of-concept and technical validation step. The study aims to provide preliminary evidence on the feasibility and accuracy of the dual-tracer protocol and is not intended to guide immediate intraoperative decision-making. The multicenter phase III SENORITA-1 randomized trial in South Korea showed that, in early GC, 5-year survival was not compromised by avoiding unnecessary lymphadenectomy in SLN-negative, treatment-naïve patients, while enabling less extensive gastrectomies and improving quality of life^8,9^.

The SLN concept is based on the premise that tumor cells initially disseminate to a specific lymph node, the SLN, before involving other regional lymph node stations^10^.

Sentinel lymph node biopsy (SLNB) is an established key tool in guiding surgical decision-making in other solid tumors, such as breast cancer and melanoma, with proven diagnostic and prognostic value. Its role in GC remains debated, particularly in the context of personalized surgical treatment aimed at reducing unnecessary extensive lymphadenectomy.

The stomach’s complex lymphatic drainage and atypical metastatic patterns (“skip metastasis”) pose challenges for precise SLN identification. Moreover, metastasis may appear even without perigastric SLN involvement, limiting mapping sensitivity.

In GC, the dual method combining radiocolloid and dye (blue or ICG) has consistently proven effective for SLN biopsy^11^. Yet, due to the high costs, potential biological risks, and limited availability of radioisotopes, we opted to use a magnetic nanoparticle tracer: superparamagnetic iron oxide (SPIO, MagTrace^®^), together with the portable SentiMag^®^ system for SLN detection. This method, combined with methylene blue, offers a safe and innovative alternative, already applied in breast cancer^12–15^.

The primary objective of this prospective study was to evaluate the diagnostic accuracy of ex vivo SLN detection in GC using a novel dual-tracer approach combining SPIO (not previously used in GC) and methylene blue. Secondary, exploratory objectives included: assessing the concordance between conventional histopathology (H&E) and One-Step Nucleic Acid Amplification (OSNA) for nodal staging; evaluating the feasibility of pooled OSNA analysis for non-sentinel lymph nodes (Non- SLN) and survival analyses, are considered exploratory and hypothesis-generating only.

This cohort study has been reported in line with the STROCSS guidelines^16^.

## Materials and methods

### Study design and setting

This was a prospective, observational and analytical study (NCT07147452), conducted at a secondary-level university hospital with 500 inpatient beds. Consecutive patients with an indication for oncological gastric surgery who met the inclusion criteria were enrolled between August 2017 and March 2022.

All patients were diagnosed, treated and followed up according to the standard clinical practice of the institution, following an evidence-based protocol aligned with the European guidelines (ESMO-ESSO-ESTRO) for the management of GC^17,18^.

### Inclusion criteria

Patients older than 18 years with histologically confirmed gastric adenocarcinoma, who underwent curative-intent surgery (total or subtotal gastrectomy) with D1, D1+, or D2 lymphadenectomy, and with preoperative positivity for cytokeratin 19 (CK19) expression in tumor samples. CK19 positivity was required because OSNA detects lymph node metastases by quantifying CK19 mRNA; ensuring that at least 30% of the primary tumor expresses this marker prevents false-negative results and thus guarantees the analytical validity of the molecular assay.

### Exclusion criteria

Patients with recurrent GC, other active malignancies, metastatic disease at diagnosis, or gastroesophageal junction carcinoma type Siewert I or II were excluded.

### Variables collected

For each patient, the following demographic, clinical, and pathological variables were systematically recorded: age, sex, tumor type, size, grade, TNM stage (8th edition), overall stage, administration of neoadjuvant treatment and tumor regression grade.

Perioperative variables included type of surgical procedure, extent of lymphadenectomy, timing of tracer injection and detection, intraoperative complications, reoperation rates, and hospital length of stay.

During follow-up, oncological outcomes were evaluated, including recurrence rate, mortality, overall survival (OS), and disease-free survival (DFS). Follow-up time was defined as OS, considering a common cut-off date for all patients of July 15, 2025.

### Surgical procedures

Study participation did not modify surgical indications or technique. The only addition was intraoperative SPIO administration, performed laparoscopically or via open surgery as appropriate.

SPIO infiltration was performed by injecting 0.5 mL of undiluted SPIO into each of the four peritumoral quadrants using an insulin-type or wingless butterfly needle suitable for laparoscopic insertion. In early GC cases with no macroscopic tumor identification, intraoperative endoscopy was used for tracer injection.

#### Sentinel lymph node detection

SLN detection was performed using an ex vivo protocol. Immediately after specimen extraction, the stomach was opened along the anterior wall, following the greater curvature, while avoiding injury to adjacent lymphatic chains.

Submucosal methylene blue injection was then performed at the tumor periphery, with 0.5 mL administered per quadrant (total 2 mL). After injection, a waiting period of 10–15 min was observed, during which gentle massage lymphatic drainage. Migration times of both tracers (methylene blue and SPIO) were recorded.

SLNs were subsequently dissected and removed if stained with methylene blue and/or detected with the pre-calibrated Sentimag^®^ probe. Nodes were immediately transported under cold conditions (0–4 °C), without preservative solution, in individual sterile containers placed on ice. For each lymph node, the anatomical station was recorded according to the Japanese Gastric Cancer Treatment Guidelines^19^. Different nodal stations (groups 1–12) were separated into independent containers according to the type of gastrectomy and extent of lymphadenectomy performed.

### Pathological processing

The surgical specimen, SLNs, and separated nodal stations were immediately delivered to the pathology department. Dissection was performed fresh under sterile conditions on a cold surface (0–4 °C). New instruments were used for each sample to prevent cross-contamination with potential mRNA residues.

The identified SLNs, were bisected into two symmetric halves: one for conventional histopathology and the other for molecular analysis using the OSNA assay.

For histology, a 1 mm longitudinal slice from the largest diameter of the designated half was placed in a cassette for H&E staining.

The remaining half was placed into a PCR tube (similar to an Eppendorf tube but specifically designed for PCR assays), kept on crushed ice, and processed by OSNA.

**Fig. 1:**
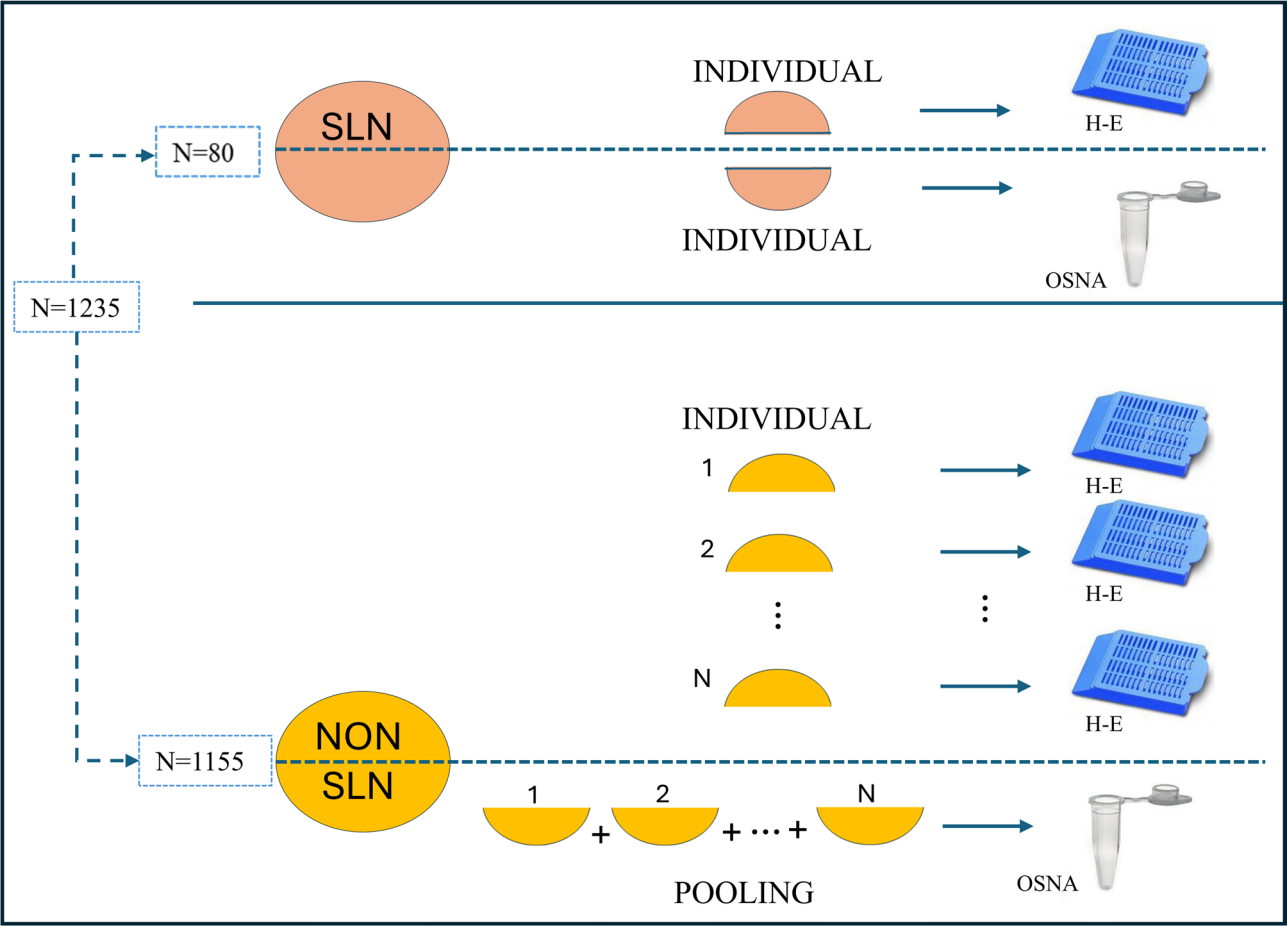
Nodal processing. Surgical lymph node processing procedure showing division and preparation for histological and molecular analysis. Non-SLN are assessed molecularly in pooled samples, while conventional histological analysis is performed individually.

The pooling analysis excludes the SLNs, which were processed individually. Non-SLN were also bisected, with one half analyzed individually by H&E and the other half subjected to molecular analysis. The portions designated for OSNA were pooled homogeneously by nodal station (pooling), with a maximum of 0.6 g of tissue per PCR tube, following protocols previously described in colorectal cancer^20^(Fig. 1).

When no discrete lymph nodes were identified in a nodal station, randomly selected adipose tissue fragments were analyzed by H&E, and the remaining tissue was assessed by OSNA pooling.

A result was considered positive if the total tumor load was ≥ 250 copies/µL. PCR tubes were processed according to the manufacturer’s protocol (Sysmex Corp., Japan), as applied in previous studies^21–24^.

Because no single gold standard exists for defining SLN status, a composite reference standard was used. An SLN was considered positive if metastatic involvement was detected by either pathological method (H&E or OSNA).

In discordant cases, OSNA-positive results, considered pathological in our study, did not alter TNM staging or subsequent treatment decisions.

To determine whether the detected SLN corresponded to the “true SLN”, the following criteria were established (in line with definitions previously used by other authors)^25–27^:

- A true SLN was defined as a positive SLN with or without positive non-SLNs (SLN+ / non-SLN±), or a negative SLN with negative non-SLNs (SLN– / non-SLN–).
- Not true SLN was considered when the SLN was negative but any non-SLN was positive (SLN– / non-SLN+).

Patients without detected SLN:

- Classified as true SLN if the entire lymphadenectomy was negative, because a hypothetical SLN (if detected) would also be negative and would meet the true SLN negative criteria.
- Classified as not true SLN if any non-SLN was positive, assuming the worst-case scenario that the undetected SLN would have been negative.

### Statistical analysis

Data were analyzed using SPSS version 26.0 (IBM Corp., Armonk, NY, USA) and EpiDat version 4.2 (Xunta de Galicia, Spain). Descriptive analyses were performed using measures of central tendency and dispersion for quantitative variables and absolute/relative frequencies for qualitative variables.

The chi-square test with correction was used to compare qualitative variables Agreement between methods was assessed using Cohen’s kappa coefficient. Concordance was evaluated per patient, comparing OSNA pooling results with dichotomized H&E findings (positive if any node was positive). Stations analyzed exclusively by OSNA were excluded. Diagnostic accuracy was estimated by sensitivity and specificity. For survival analyses, early postoperative mortality (defined as death within 90 days after surgery) was excluded to minimize confounding by perioperative factors unrelated to tumor biology. Survival was analyzed using Kaplan–Meier curves and the log-rank test. Prognostic independence of OSNA and H&E was assessed using multivariable Cox regression. A p-value < 0.05 was considered statistically significant. Analyses were considered exploratory due to the limited sample size and small number of events.

The study was a prospective feasibility investigation with consecutive patient enrollment. The sample size was pragmatically determined based on the expected number of gastric adenocarcinoma cases with lymph node involvement (*p* = 0.75) within a finite population of 40 patients during the study period. Using a standard formula with finite population correction, the minimum required sample size was estimated to be 37 patients. The study was primarily designed to evaluate diagnostic accuracy outcomes related to SLN detection. It was not specifically powered for survival or multivariable analyses. Accordingly, these results should be interpreted as exploratory and hypothesis-generating.

## Results

### Sample characteristics

**Fig. 2:**
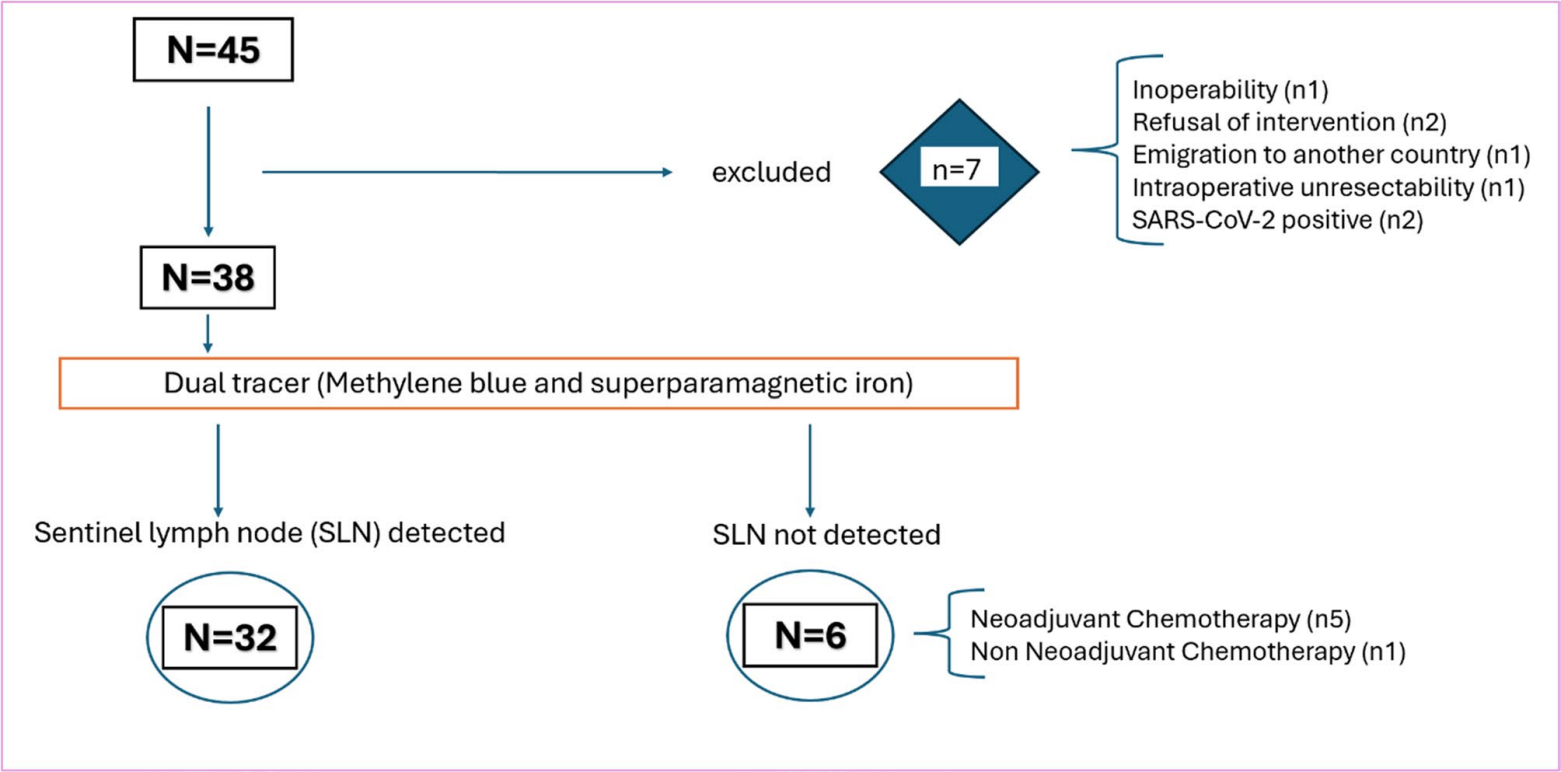
Flow diagram of study participants. Flowchart illustrating the selection, inclusion, and exclusion of study participants, including numbers at each stage of the trial.

A total of 45 patients who met general eligibility criteria were initially evaluated for inclusion in the study. An additional two patients were screened but were not considered for inclusion due to insufficient CK19 expression (4.3% of the screened population), consistent with published data indicating that CK19-negative gastric adenocarcinomas are uncommon^28^. Of the 45 patients, 7 were subsequently excluded for other reasons (see Fig. 2), leaving 38 patients who were enrolled in the study, all of whom underwent SLN detection using the dual-tracer protocol (SPIO and methylene blue). SLN identification was successful in 32 cases.

The mean age was 64.3 ± 9.6 years; 57.9% (*n* = 22) were male and 42.1% (*n* = 16) were female.

(Table 1) summarizes the main pathological features.

**Table 1:** Pathological characteristics of study participants.

Variable	Mean ± SD/ *n* (%)
Tumor size (cm) *	2.48 ± 1.70
Lauren classification	
Intestinal	20 (52.6)
Difusse	15 (39.5)
Mixed	3 (7.9)
Tumor location	
Cardia	5 (13.2)
Fundus	1 (2.6)
Gastric body	5 (13.2)
Antrum	23 (60.5)
Prepyloric	4 (10.5)
Degree of differentiation	
G1	6 (15.8)
G2	15 (39.5)
G3	17 (44.7)
Lymphovascular invasion	13 (34.2)
Perineural invasion	14 (36.8)
Type of resection	
R0	35 (92.1)
R1	3 (7.9)
Neoadjuvant treatment	27 (71.1)
Tumor regression (TRG)	
TRG 1	3 (7.9)
TRG 2	4 (10.5)
TRG 3	9 (23.7)
TRG 4	4 (10.5)
TRG 5	7 (18.4)
Pathological T stage (TNM th ed.)	
T0	3 (7.9)
Tis	5 (13.1)
T1a	1 (2.6)
T1b	5 (13.1)
T2	7 (18.4)
T3	11 (28.9)
T4a	5 (13.1)
T4b	1 (2.6)
Pathological N stage (TNM 8th ed.)	
N0	23 (60.5)
N1	5 (13.1)
N2	6 (15.8)
N3a	3 (7.9)
N3b	1 (2.6)
Clinical Stage (AJCC 8th ed.)	
0	8 (21.1)
IA	6 (15.8)
IB	3 (7.9)
IIA	8 (21.1)
IIB	1 (2.6)
IIIA	7 (18.4)
IIIB	3 (7.9)
IIIC	2 (5.3)

Data are presented as mean ± standard deviation (SD) or number (percentage). R0 = microscopically margin-negative resection; R1 = microscopically margin-positive resection; G1–G3 = histological differentiation grade (well, moderate, poor); TRG = tumor regression grade according to the College of American Pathologists system; TNM and clinical stage classified according to the 8th edition of the AJCC/UICC. (*) Tumor size refers to pathological measurement, often post-neoadjuvant.

### Surgical data and techniques

Among the 38 gastrectomies performed, 29 (76.3%) were subtotal and 9 (23.7%) were total. A laparoscopic approach was used in 29 patients (76.3%). With respect to lymphadenectomy, D2 dissection was performed in 33 patients (86.8%), D1 + in 3 patients (7.9%), and D1 in 2 patients (5.3%) due to comorbidities. Mean hospital stay was 10.0 ± 8.9 days, with a median of 8 days (range 5–60).

Both tracers were administered in all patients. The mean time from injection to detection was 196 ± 56 min for SPIO and 32 ± 8 min for methylene blue.

### SLN detection rate and tracer accuracy

SLNs (*n* = 80) were identified in 32 of 38 patients (84.2%). In the six patients without SLN detection (4 men, 2 women; mean age 64.3 years), tumor size ranged from 0.8 to 7 cm and tumors were predominantly located in the antrum. Three tumors were intestinal type and three diffuse type; diffuse histology and radial margin involvement were more frequent among non-detected cases. Most patients (83.3%) had received neoadjuvant chemoradiotherapy. Metastases were detected by OSNA in two patients, one of whom was also positive by H&E, indicating that failure to identify an SLN did not preclude nodal involvement.

A total of 80 SLNs were analyzed: 23 were positive only for methylene blue, 26 for SPIO, and 31 for both tracers. The mean number of SLNs per patient was 2.11 ± 1.35 (range 0–4). Diagnostic performance of the dual-tracer method was evaluated using a 2 × 2 contingency table, which explicitly reports concordant and discordant classifications relative to true SLN. Sensitivity, specificity, and predictive values were subsequently calculated from these raw data and are summarized in Table 2. Confidence intervals were wide for specificity and negative predictive value, reflecting the limited sample size and the small number of true-negative (TN) cases.

**Table 2:** Diagnostic performance of the dual-tracer technique for true SLN detection based on per-patient analysis.

	True SLN *	Not true SLN**	Total
Dual-tracer (+)	27 (TP)	5 (FP)	32
Dual-tracer (-)	2 (FN)	4 (TN)	6
Total	29	9	38

Parameter		Value (%)	95% CI
Sensitivity		93.10	82.16–100
Specificity		44.44	6.43–82.46
Positive predictive value (PPV)		84.38	70.23–98.52
Negative predictive value (NPV)		66.67	20.61–100
Accuracy		81.58	67.94–95.22
Prevalence		76.32	61.48–91.15

*True SLN was defined as SLN+ with or without positive non-SLNs (SLN+ / non-SLN±), or SLN– with negative non-SLNs (SLN– / non-SLN–).

**Not true SLN was defined as SLN– with any positive non-SLN (SLN– / non-SLN+). In patients without detected SLN, cases were classified as true SLN if all non-SLNs were negative, and as not true SLN if any non-SLN was positive. Lymph nodes were considered positive according to the established composite standard, defined as metastatic involvement detected by OSNA and/or H&E.

Specificity reflects agreement with this composite reference standard and does not represent histopathological or molecular specificity at the nodal level.

Pearson’s chi-square test with Yates’ correction showed a significant association between tracer-based classification and histopathology (χ² = 4.732; *p* = 0.029). Concordance between tracers was moderate, with a Cohen’s kappa of 0.4242 (95% CI: 0.0755–0.7730) and an observed agreement of 81.6% (*p* = 0.007).

When SLNs were compared with the remaining lymphadenectomy, 27 of 32 patients with detected SLNs (84.4%) corresponded to the true SLN, whereas 5 cases (15.6%) were classified as not true SLN (see Table 3). OSNA-positive findings did not modify TNM staging or subsequent therapeutic decisions.

**Table 3:** Distribution of true and not true SLN status in patients with SLN detected using the dual-tracer technique (*n* = 32).

True SLN status	SLN status	Non-SLN status	*n*
True SLN positive	SLN+	Non-SLN+	2
	SLN+	Non-SLN–	9
True SLN negative	SLN–	Non-SLN–	16
Not true SLN	SLN–	Non-SLN+	5

True SLN includes both True SLN positive and True SLN negative.

### Per-patient analysis of concordance between H&E and OSNA in the entire lymphadenectomy

A total of 1235 lymph nodes, including SLNs, were examined, with a mean of 32.5 ± 19.42 nodes per patient (range 12–105) 0.82 nodes were metastatic, with a mean of 2.16 ± 5.17 affected nodes per patient (range 0–29). The proportion of patients with metastatic nodes on H&E was 39.5% (15/38), increasing to 47.4% when considering positivity by either H&E or OSNA (18/38).

Diagnostic performance using H&E as the gold standard is shown in (Table 4). The same results were obtained when OSNA was assumed as the reference standard, due to the symmetrical distribution of discordant cases.

**Table 4:** Diagnostic performance of OSNA versus reference H-E test for patient-based overall nodal status (*n* = 38).

	H&E Negative	H&E Positive	Total
OSNA Negative	20	3	23
OSNA Positive	3	12	15
Total	23	15	38

Parameter		Valor (%)	95% CI
Sensitivity		80.00	56.42–100
Specificity		86.96	71.02–100
Positive predictive value (PPV)		80.00	56.42–100
Negative predictive value (NPV)		86.96	71.02–100
Accuracy		84.21	71.30–97.12
Prevalence		39.47	22.62–56.33

Regarding discordant cases, six patients (15.8%) showed discrepant results between OSNA and H&E. Three patients were OSNA-positive but H&E-negative, whereas three patients were H&E-positive but OSNA-negative, resulting in a symmetrical distribution of discordances. This symmetrical crossover explains why diagnostic performance estimates remained identical regardless of which technique was considered the reference standard.

The concordance analysis between H&E and OSNA, assuming H&E as the reference standard, demonstrated a Cohen’s kappa of 0.6696 (95% CI: 0.4276–0.9115), with statistical significance confirmed by Fisher’s exact test (*p* < 0.001).

### Concordance of pooling analysis in Non-SLN

When concordance was analyzed for non-SLN (*n* = 1155), comparing individual H&E results (node-by-node) with OSNA findings by lymph node station, agreement per patient was moderate to strong in most groups (3, 4, 6–8) and perfect in Group 9, with statistically significant concordance (Table 5). Weak or non-significant agreement was observed in Groups 5 and 12, mainly due to the presence of lymphatic tissue without identifiable lymph nodes in two patients per group, which showed micro- or macrometastases by OSNA that could not be evaluated by H&E. On subsequent review, these cases corresponded to lymphangitic carcinomatosis.

**Table 5:** Agreement between individual H&E and pooled OSNA for Non-SLN grouped by nodal station.

	Kappa	95% CI	Type of Agreement
1	0.475	0.029–0.927	Moderate
2	—	—	—
3	0.465	0.163–0.767	Moderate
4	0.529	0.192–0.866	Moderate
5	0.242	−0.222–0.706	Weak
6	0.713	0.456–0.970	Strong
7	0.642	0.152–1.000	Strong
8	0.775	0.426–1.000	Strong
9	1.000	1.000–1.000	Perfect
11	—	—	—
12	0.455	−0.047–0.957	Moderate

Groups 2 and 11 could not be compared due to one of the techniques being consistently negative.

### Comparative analyses (Neoadjuvant therapy and true SLN detection)

In the exploratory subgroup analysis, sensitivity and negative predictive value may suggest preserved feasibility in the neoadjuvant subgroup (*n* = 27), with sensitivity of 95% (95% CI: 82.95–100) and NPV of 80% (95% CI: 34.95–100). In the non-neoadjuvant subgroup (*n* = 11), sensitivity 100% (95% CI: 94.44–100) and NPV 100% (95% CI: 50.0–100). These findings should be interpreted with caution due to small subgroup sizes and wide confidence intervals. No definitive conclusions can be drawn, and the results are considered hypothesis-generating.

### Follow-up outcomes

For survival analysis, 3 patients who died early from surgical complications were excluded, leaving 35 patients.

Median overall survival (OS) was 55 months (IQR 35–72). Mean disease-free survival (DFS) was 47.4 ± 27.3 months.

Recurrence occurred in 40% of patients, either locoregional or distant, with a mean time to recurrence of 19.3 ± 10.3 months.

The overall mortality rate, including the three perioperative deaths, was 39.5%, with a mean follow-up of 53 ± 24.4 months.

Survival outcomes (OS and DFS) are reported for exploratory purposes only; no definitive conclusions can be drawn due to the limited sample size and study design.

Kaplan–Meier survival analysis: OSNA-negative patients showed longer survival (81.01 months; 95% CI: 69.36–92.66) than OSNA-positive patients in univariable analysis (*p* = 0.017). Similarly, H&E-negative patients had higher survival (82.44 months; 95% CI: 71.94–92.94) compared with H&E-positive patients (*p* = 0.007). In multivariable Cox regression adjusted for TNM stage, OSNA lost significance (*p* = 0.253) but remained significant for neoadjuvant therapy (*p* = 0.021) and margin status (*p* = 0.034). H&E showed a similar pattern.

## Discussion

### Interpretation of the results

In this study, ex vivo lymphatic mapping of GC using a dual-tracer approach (magnetic nanoparticles and methylene blue) enabled identification of the true SLN in most patients, with moderate agreement between histological and molecular (OSNA) assessments.

These findings demonstrate technical feasibility and methodological concordance under controlled ex vivo conditions and should be interpreted strictly within this experimental framework.

Low specificity in the ex vivo setting may result from non-specific tracer diffusion, spatial allocation bias due to node splitting, and methodological differences between OSNA and H&E, particularly in nodes with micrometastases.

Regarding survival outcomes, in univariable analyses, positive OSNA status was associated with shorter survival, reflecting its correlation with tumor burden and disease extent. However, after adjustment for TNM stage in multivariable models, OSNA was no longer independently significant. This finding could suggest that OSNA status acts as a surrogate marker of tumor burden and disease extent, rather than as an independent prognostic factor. The association between OSNA results and established staging parameters indicates that the prognostic information provided by OSNA substantially overlaps with that captured by TNM classification. Consequently, OSNA would appear to be primarily useful as a methodological staging tool within a research setting, rather than as a marker providing additional prognostic information beyond conventional staging. Observed associations with survival must be interpreted cautiously and considered hypothesis-generating, as the study was not powered for these analyses.

### Comparison with previous studies

Comparisons with other studies should be interpreted cautiously given the considerable methodological heterogeneity among the main investigations. The major sources of variability include: tumor stage, timing of mapping, number and type of tracers used, unit of calculation for true positives and negatives (per patient or per node), criteria for defining the true SLN, reference standard (H&E, OSNA, or both), and whether the analysis was intraoperative or deferred^21–24,29–31^.

Our results show that the dual tracer method offers adequate and reproducible methodological performance in identifying the SLN in GC, under *ex vivo* conditions, supporting its technical validity. Although the observed performance did not reach excellent levels (likely related to the limited sample size) it was acceptable and consistent with published evidence. Current data support the use of dual tracers (radiocolloid and blue dye/ICG), with proven efficacy in both submucosal and subserosal injections provided they are administered in the peritumoral area^11,25,30^, while European studies using a single tracer have proven ineffective^21^. SPIO emerges as a potential alternative, avoiding the use of radioisotopes while maintaining adequate sensitivity beyond 20 min after administration^32^, unlike what has been reported with tracers such as indocyanine green^33^.

The OSNA assay showed acceptable analytical performance for nodal assessment in this cohort, with a balanced sensitivity and specificity. As expected, neither parameter reached perfect values. The relatively high negative predictive value (NPV 87%) suggests that, under controlled ex vivo conditions, negative OSNA findings were consistent with the absence of metastatic involvement in sentinel lymph nodes. Similar diagnostic properties of OSNA have been reported in early-stage GC in intraoperative settings, such as in the study by Shimada et al.^22^. In the present study, these findings should be interpreted strictly as technical validation, and any potential role of OSNA in intraoperative decision-making would require dedicated prospective in vivo studies.

The meta-analysis by Wang et al.^30^ reported a very wide range of FN rates (2–59%), largely conditioned by the gold standard used. It has also been suggested that results can be optimized by applying grouped sensitivity, i.e., considering the combined positivity of both techniques as the reference. In our series, the FN rate reached 20%, regardless of whether OSNA or H&E was used as the reference standard. This finding may be partly influenced by methodological factors inherent to the ex vivo setting, including tissue allocation bias and RNA degradation related to freezing and deferred molecular analysis. While previous studies have suggested that OSNA performance may benefit from real-time processing, the present results should be interpreted strictly within the limitations of the ex vivo design and do not allow conclusions regarding comparative intraoperative performance.

The JCOG0302 study reported an unexpectedly high FN rate of 46% in intraoperative H&E analysis, leading to premature trial suspension. This high rate was attributed mainly to an insufficient learning curve and methodological limitations, as only a single frozen section of each lymph node was analyzed. Although multiple frozen sections could be examined intraoperatively, this is time-consuming and still associated with an unacceptable FN rate^34^.

Our elevated FN rate may be partly attributable to the division of each lymph node between the two techniques. Furthermore, the limited sensitivity of H&E for detecting micrometastases must be highlighted, particularly compared with OSNA, which shows superior ability to detect microscopic subclinical disease^22,35–37^. Discordant results between OSNA and H&E should be interpreted with caution, as they may partly reflect tissue allocation bias inherent to splitting each lymph node (see Limitations).

The efficacy of OSNA for detecting nodal metastases in GC has been demonstrated in prior studies conducted in Japan; whereas, evidence supporting its utility in Western settings remains scarce^21–24,31^.

The incorporation of OSNA into this protocol represents an improvement over purely histological methods under controlled ex vivo conditions. Unlike conventional techniques, OSNA provides objective, consistent, and rapid results (within 30–40 min), with the added ability to process up to 14 samples simultaneously in its updated versions.

The Korean SENORITA 2 trial, a prospective multicenter phase II study, evaluated the technical feasibility of dual tracer mapping and the SLN detection rate in patients with early GC who had already undergone a prior non-curative endoscopic submucosal resection. This study is particularly relevant, as it showed that fibrosis or tumor involvement may alter lymphatic drainage pathways, resulting in tracer deviations and subsequent FN results in SLN mapping—an effect also observed in patients who have received neoadjuvant therapy^38^. In this context, our exploratory subgroup analysis suggests that SLN mapping remains feasible following neoadjuvant treatment, although diagnostic performance appeared less favorable than in the non-neoadjuvant cases, which showed optimal sensitivity and NPV. However, given the small subgroup sizes and the descriptive nature of these analyses, these observations should be regarded as hypothesis-generating only and not used to guide patient selection or infer clinical performance. These findings remain preliminary and require validation in larger prospective studies.

It is worth noting that SLN non-detection in certain cases in our series could be attributed to advanced tumor stages with prior neoadjuvant treatment, except for one patient who did not present these features, as has also been described in other studies^39^. In general, tumors staged as cT3–4, which constitute the majority of our patient population, are not considered ideal candidates for SLN biopsy according to the literature, due to their higher likelihood of extensive nodal spread. Importantly, most published studies on this technique have been conducted in early-stage disease^9^.

In our study, when no lymph nodes were identified in a given station, the surrounding lymphatic-fatty tissue was analyzed with OSNA. In some cases, this revealed micrometastases, also known as extranodal extension (ENE). These observations were associated with cases showing carcinomatous lymphangitis on H&E, advanced stages, and worse disease-free survival. These findings should therefore be regarded as exploratory and hypothesis-generating, likely related to the small number of events and the unconventional nature of the tissue analyzed. Rather than constituting a confirmed diagnostic entity, they may raise the hypothesis of perinodal tumor spread without overt nodal involvement, potentially associated with adverse prognostic features. Similar associations have been reported in previous studies based on histopathological assessment alone, without molecular analysis^40–42^.

We also highlight the use of pooling for non-SLN, which optimizes resources (time and cost) and avoids subsampling bias, as occurred in the Shimada et al. study^22^. Owing to the intrinsic characteristics of the OSNA technique, pooled positive results cannot be retrospectively resolved to individual lymph nodes, as the analyzed tissue is completely homogenized and consumed during the assay. In total, we analyzed 1155 lymph nodes (non-SLN), which represents a substantial sample size and a novel aspect of our study, particularly in combination with the use of SPIO, as no similar studies in GC have yet been published. Rakislova et al.^20^ applied a similar pooling approach for OSNA in colorectal cancer to improve efficiency. Since then, technical developments have enabled the analysis of a larger number of samples than was possible at the time of our study. Combined analysis allows evaluation of multiple lymph nodes with generally high concordance; although, weaker agreement was observed in certain nodal stations. These findings may reflect underlying anatomical or biological heterogeneity rather than technical failure and therefore warrant a more nuanced interpretation. Consequently, although pooled approaches may facilitate broader nodal assessment, further refinement of staging systems may be required to incorporate measures of tumor burden beyond the simple count of involved nodes^43,44^. Accordingly, pooled OSNA should not be interpreted as a substitute for conventional histopathological nodal staging but rather as a methodological strategy for research-oriented ex vivo assessment.

### Strengths of the study

The key strength of this study lies in its prospective design and the cross-validation between conventional histology and molecular analysis, which reinforces the technical robustness of SLN detection. The combined use of tracers provided both qualitative and quantitative redundancy, facilitating SLN identification even in anatomically complex cases. In addition, the follow-up period allowed us to assess not only immediate diagnostic performance but also longer-term oncologic outcomes, such as disease-free survival, although the sample size limits definitive conclusions.

### Limitations

This study has several limitations, including a moderate sample size and its single-center design. All analyses were conducted under ex vivo conditions, so the findings cannot be directly extrapolated to intraoperative practice and should be interpreted as a technical feasibility and methodological validation only.

SLN biopsy is less validated in advanced-stage GC, which may limit the generalizability of our findings in this subgroup. Another limitation is the allocation of different tissue fractions from the same lymph node to OSNA and H&E analysis, which introduces tissue allocation bias, particularly in low-volume disease such as micrometastases. Discordant results may therefore reflect spatial heterogeneity of tumor deposits rather than true methodological disagreement, a limitation inherent to comparative molecular–histological studies.

A composite reference standard was used to define true SLN status, as no single gold standard exists; therefore, incorporation bias cannot be entirely excluded. Pooled analysis of non-SLN can detect the presence of metastases but does not identify which individual nodes are affected, limiting precise nodal staging.

Finally, stratified analyses were limited by small subgroup sizes or absence of events, which reduces statistical power and may increase the risk of estimation bias. In addition, the limited sample size and small number of events may have resulted in instability of multivariable Cox regression estimates and potential overfitting. Therefore, these survival analyses should be interpreted as exploratory and hypothesis-generating rather than confirmatory.

### Clinical implications and future research

From a translational perspective, the results of this study should be regarded strictly as hypothesis-generating. While ex vivo dual-tracer SLN mapping and molecular nodal assessment were feasible under controlled conditions, the observed limitations in specificity indicate that the current data do not support changes in surgical strategy or intraoperative decision-making. Observed associations with survival are exploratory and hypothesis-generating only. Prospective in vivo validation in larger, homogeneous cohorts is required to confirm reproducibility and diagnostic performance before any potential clinical relevance can be evaluated.

## Conclusions

This study suggests the technical feasibility of ex vivo SLN mapping in GC using a dual-tracer approach combining SPIO and methylene blue. The integration of OSNA with conventional histology showed acceptable methodological concordance for nodal assessment, and pooled molecular analysis allowed efficient evaluation of non-SLN within an ex vivo experimental framework. These findings constitute a technical and methodological validation. Observed associations with survival should be interpreted as exploratory and hypothesis-generating. Larger prospective in vivo studies are required before any clinical application can be considered.

## Data Availability

The data supporting the findings of this study are available from the corresponding author upon reasonable request.
